# Nicotine and novel tobacco products drive adverse cardiac remodeling and dysfunction in preclinical studies

**DOI:** 10.3389/fcvm.2022.993617

**Published:** 2022-10-06

**Authors:** Nicholas D. Fried, Joshua M. Oakes, Anna K. Whitehead, Eric Lazartigues, Xinping Yue, Jason D. Gardner

**Affiliations:** ^1^Department of Physiology, Louisiana State University Health Sciences Center, New Orleans, LA, United States; ^2^Department of Pharmacology & Experimental Therapeutics, Louisiana State University Health Sciences Center, New Orleans, LA, United States; ^3^Cardiovascular Center of Excellence, New Orleans, LA, United States; ^4^Neuroscience center of Excellence, Louisiana State University Health Sciences Center, New Orleans, LA, United States; ^5^Southeast Louisiana Veterans Health Care Systems, New Orleans, LA, United States

**Keywords:** nicotine, electronic cigarettes (E-cigarettes), heat-not-burn (HNB), cardiac remodeling, novel tobacco products

## Abstract

**Background:**

The heart undergoes structural and functional changes in response to injury and hemodynamic stress known as cardiac remodeling. Cardiac remodeling often decompensates causing dysfunction and heart failure (HF). Cardiac remodeling and dysfunction are significantly associated with cigarette smoking. Although cigarette smoking has declined, the roles of nicotine and novel tobacco products (including electronic cigarettes and heat-not-burn tobacco) in cardiac remodeling are unclear. In this perspective, we present evidence demonstrating maladaptive cardiac remodeling in nicotine-exposed mice undergoing hemodynamic stress with angiotensin (Ang)-II infusion and review preclinical literature linking nicotine and novel tobacco products with cardiac remodeling and dysfunction.

**Methods:**

Adult, male C57BL/6J mice were exposed to room air or chronic, inhaled nicotine for 8 weeks. A subset of mice was infused with Ang-II *via* subcutaneous osmotic mini-pumps during the final 4 weeks of exposure. Left ventricular structure and function were assessed with echocardiography.

**Results:**

Chronic, inhaled nicotine abrogated Ang-II-induced thickening of the left ventricular posterior wall, leading to reduced relative wall thickness. Ang-II infusion was associated with increased left ventricular mass index in both air- and nicotine-exposed mice.

**Conclusions:**

These changes suggest a phenotypic shift from concentric hypertrophy to eccentric hypertrophy in nicotine-exposed, hemodynamically-stressed mice which could drive HF pathogenesis. These findings join a growing body of animal studies demonstrating cardiac remodeling and dysfunction following nicotine and electronic cigarette exposure. Further exploration is necessary; however, clinicians and researchers should not overlook these emerging products as potential risk factors in the pathogenesis of cardiac remodeling and associated diseases including HF.

## Introduction

Following injury or hemodynamic stress, the heart undergoes extensive structural and functional changes known collectively as cardiac remodeling (1). The mechanisms driving cardiac remodeling are complex and involve cellular death, inflammation, oxidative stress, modified energy and calcium homeostasis, neurohormonal activation, altered contractile machinery, fibrosis and extracellular matrix remodeling, and cardiomyocyte hypertrophy with geometric changes (2). Cardiac remodeling is concerning due to its propensity to decompensate toward cardiac dysfunction and disease states including myocardial infarction and heart failure (HF) (2, 3). Cardiac remodeling, cardiac dysfunction, and HF are significantly associated with both current and former cigarette smoking, as well as second-hand smoke exposure (4–11). Although cigarette smoking has declined worldwide (12), the cardiovascular risks of using novel tobacco products, including electronic cigarettes (13–15) and heat-not-burn tobacco (16), are poorly established.

Electronic cigarettes generate vapor by heating liquid containing nicotine, vegetable glycerin, propylene glycol, flavoring additives, and other chemicals. In contrast, heat-not-burn tobacco products generate nicotine-containing vapor by heating a cigarette-like tobacco plug to sub-combustion temperatures; this process aims to provide a similar experience to cigarette smoking while reducing exposure to toxic inhalants (17). Studies comparing nicotine emissions between electronic cigarettes, heat-not-burn tobacco products, and combustible cigarettes have been inconclusive (18–21). Clinically, differences in nicotine delivery between various products are likely insignificant due to nicotine dose titration by consumers (22).

A recent cohort study of over 5 million Korean adults found that short-term cardiovascular disease risk was reduced in patients who switched from cigarette smoking to novel tobacco product use (23). While this finding is promising from a public health perspective, patients switching from cigarette smoking to novel tobacco product use had 1.7 times greater risk of developing cardiovascular disease in comparison to patients who quit nicotine and tobacco products entirely (23). Due to concerns surrounding the shifting nicotine and tobacco consumption landscape, representatives of the American Heart Association, World Heart Federation, American College of Cardiology, and European Society of Cardiology issued a joint statement calling for further study of the cardiovascular health effects of nicotine and novel tobacco product use (24).

Despite the well-established relationship between cigarette smoking and cardiovascular pathology, the roles of nicotine and novel tobacco product use in these conditions are unclear. Epidemiological studies of novel tobacco products currently face challenges including the long time-course of cardiovascular disease pathogenesis and confounding caused by previous and current use of other tobacco products in study subjects (25). One analysis of Waves 1 through 5 (2013-2019) of the Population Assessment of Tobacco and Health (PATH) Study found reduced risk of HF, myocardial infarction, and stroke in exclusive electronic cigarette users vs. combustible tobacco users (26); a second analysis of this cohort found no differences in cardiovascular disease events between combustible tobacco users, those who transitioned to electronic cigarette use or dual-use, and those that quit tobacco products (27). Authors of both studies, however, indicate significant limitations surrounding short follow-up duration in relation to the disease endpoints (26, 27). Other clinical trials using acute endpoints, which have been limited by small sample sizes, have noted potential risk factors for cardiac remodeling and dysfunction including elevated systolic and diastolic blood pressure, vascular stiffness, endothelial dysfunction, oxidative stress, and pro-thrombotic effects with the use of electronic cigarettes containing various nicotine concentrations (14). Notably, a previously healthy 19-year-old patient developed signs and symptoms of acute cardiac dysfunction during an episode of electronic cigarette or vaping use-associated lung injury (28). Small clinical studies have also identified elevated blood pressure, vascular stiffness, oxidative stress, and acute impairment of systolic and diastolic cardiac function in users of heat-not-burn tobacco (29–31).

A growing body of preclinical literature implicates nicotine and novel tobacco products in cardiac remodeling and dysfunction, highlighting concerning connections which have not been identified in early clinical studies of these devices. In this Perspective, we discuss that mounting preclinical evidence and present original data demonstrating maladaptive cardiac remodeling driven by chronic, inhaled nicotine in a mouse model.

## Materials and methods

### Animals and exposure model

Adult, male C57BL/6J mice (8–12 weeks old) from Jackson Laboratory (Bar Harbor, ME) were housed in a temperature (21 C) and humidity-controlled facility under a 12-h dark/light cycle, fed standard mouse chow (iOS Teklab Extruded Rodent Diet 2019S; Envigo, Huntingdon, United Kingdom) and water ad libitum. Nicotine-exposed mice were housed in a nicotine inhalation chamber (La Jolla Alcohol Research, La Jolla, CA), while air-exposed mice were housed outside of the chamber in the same room. All procedures were approved by the Louisiana State University Health Sciences Center Institutional Animal Care and Use Committee (#3674).

Nicotine vapor was produced by bubbling air through a gas-washing bottle containing free base nicotine (Sigma-Aldrich, St. Louis, MO), as previously described (32, 33). The concentrated nicotine vapor was then diluted with air and evenly distributed to each chamber at a flow rate of 7 to 8 L/min. Flow rate was adjusted to achieve plasma cotinine levels (approximately 600 ng/mL), comparable to those observed in human combustible cigarette and novel tobacco product users (34–36). Nicotine exposure followed a 12 h on/12 h off schedule, overlapping with the dark cycle. After 4 weeks of exposure, a subset of mice was implanted with subcutaneous osmotic mini-pumps (Alzet Model 1004; Durect Corporation, Minneapolis, MN) containing angiotensin-II (Ang-II, Sigma-Aldrich) for infusion at a dose of 450 ng/kg per min for a duration of 4 weeks while maintaining either nicotine- or air-exposure.

### Echocardiography and analysis

B-mode and M-mode echocardiographic assessment was performed using the Vevo 3,100 Imaging System with a 30-MHz probe (VisualSonics, Toronto, Canada) at the conclusion of the 8-week exposure period under 1–1.5% isoflurane anesthesia. Heart rate was maintained between 450 and 550 beats per min. Ultrasound images were processed using the leading-edge technique in Vevo LAB on a minimum of 3 cardiac cycles to calculate group averages. Corrected left ventricular (LV) mass was calculated as 0.8424 x [(LVID;d + LVPW;d + LVAW;d)^3^–LVID;d^3^], where LVID;d was LV internal diameter at diastole, LVPW;d was LV posterior wall thickness at diastole, and LVAW;d was LV anterior wall thickness at diastole. Systolic measurements are denoted as LVID;s and LVPW;s. LV mass index (LVMI) was calculated by dividing corrected LV mass by tibia length at 8 weeks. Relative wall thickness (RWT) was calculated as 2 x (LVPW;d / LVID;d).

Data are displayed as mean ± SEM. Findings were analyzed by two-way ANOVA using GraphPad Prism 9 (GraphPad Software, San Diego, CA). A Tukey–Kramer *post hoc* test for multiple comparisons between means was performed when interactions were present on two-way ANOVA. *P* < 0.05 was considered statistically significant.

### Chronic, inhaled nicotine drives maladaptive cardiac remodeling in hemodynamically-stressed mice

Cardiac dysfunction, eventually progressing to HF, occurs as a result of poor compensation following injury (such as myocardial infarction) or hemodynamic stress (such as volume or pressure overload). Cardiac hypertrophy is one mechanism that maintains cardiac function following exposure to these stresses. The compensatory phase of cardiac hypertrophy can be described using LaPlace's Law, which states that:


(1) $$\begin{array}{l} Wall\;Stress=\;\frac{Pressure\;x\;Radius}{2\;x\;Wall\;Thickness}\; \end{array}$$

In this theoretical model, parallel deposition of sarcomeres in a process called concentric hypertrophy increases LV wall thickness and decreases chamber diameter, which normalizes wall stress in response to elevated LV pressure (37). In response to increased volume, the LV walls may become thinned through series deposition of sarcomeres with dilatation of the LV chamber in a process called eccentric hypertrophy (37). Hypertrophic changes eventually become maladaptive, however, leading to impaired function and worsened pathology (37, 38). The mechanisms underlying transition from adaptive cardiac remodeling to maladaptive cardiac remodeling are poorly understood and remain an active area of research with potential ramifications in the treatment of cardiac hypertrophy and HF (39).

**Figure 1:**
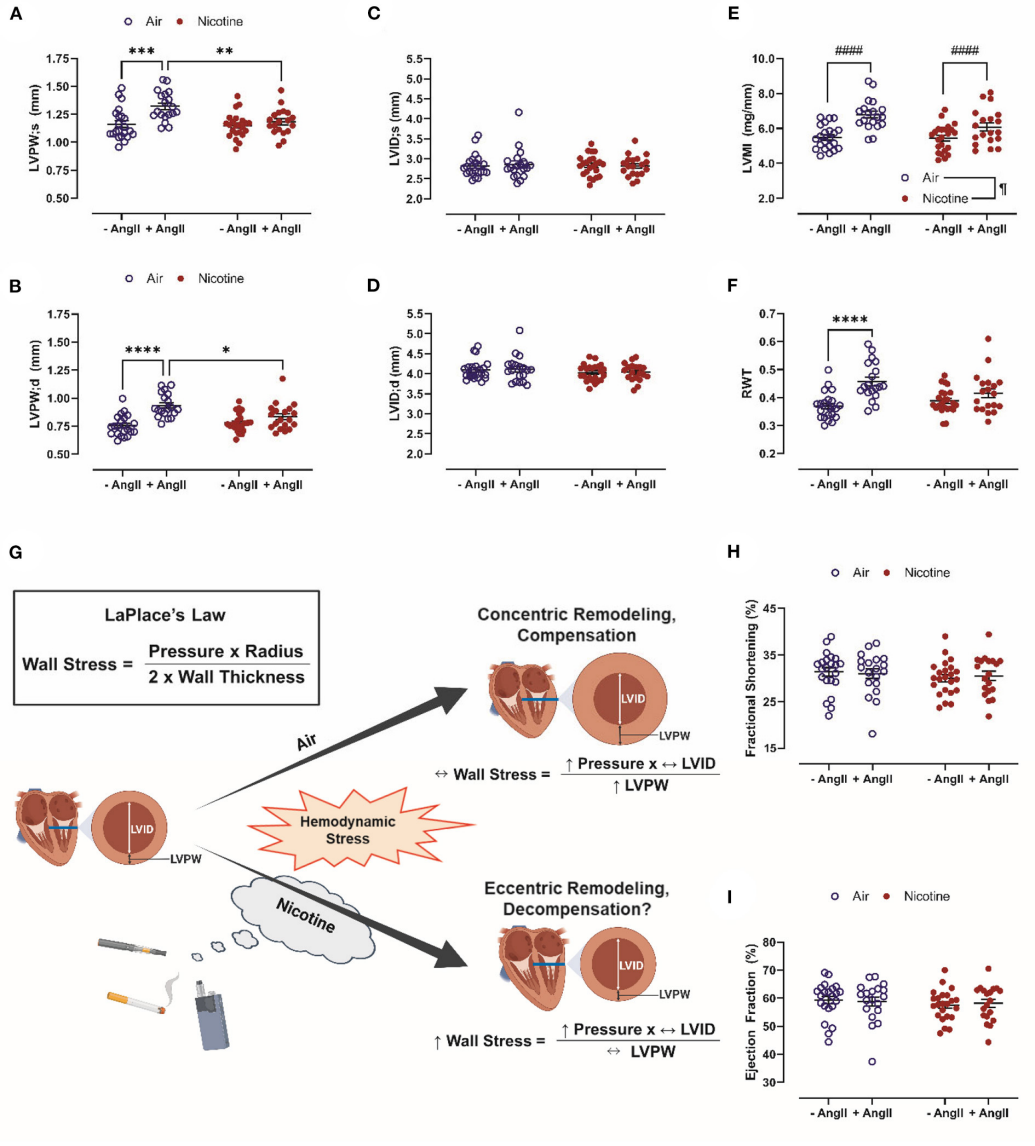
Chronic, inhaled nicotine drives maladaptive cardiac remodeling in mice undergoing hemodynamic stress. **(A,B)** Inhaled nicotine abrogates angiotensin (Ang)-II-induced left ventricular (LV) posterior wall thickening at systole and diastole (LVPW;s and ;d). **(C,D)** Neither Ang-II nor nicotine inhalation affect LV chamber diameter (LVID;s and ;d). **(E)** Ang-II increases LV mass indexed to tibia length (LVMI). **(F)** Ang-II increases relative wall thickness (RWT) in air-exposed mice only. **(G)** Inhaled nicotine exposure causes a shift from concentric to eccentric hypertrophy during hemodynamic stress which may exacerbate LV wall stress and promote decompensation based upon LaPlace's Law. **(H,I)** Neither Ang-II nor nicotine affect LV function measured by fractional shortening and ejection fraction. *N* = 19–23 per group. Data are displayed as mean ± SEM. *P < 0.05, **P < 0.01, ***P < 0.001, ****P < 0.0001 (two-way ANOVA with Tukey-Kramer *post hoc* test); #### indicates a main effect of Ang-II (*P* < 0.0001, two-way ANOVA); ¶indicates a main effect of nicotine (*P* < 0.05, two-way ANOVA). Figure 1G was created with BioRender.com.

In this study, C57BL/6J mice were exposed to room air or inhaled nicotine for 4 weeks. A subset of mice was infused with Ang-II *via* osmotic mini-pumps during an additional 4-weeks exposure period. LVPW;s (1.32 ± 0.03 mm, *n* = 19) and LVPW;d (0.93 ± 0.02 mm, *n* = 19) in Air + Ang-II mice were significantly increased vs. both Air mice (LVPW;s: 1.16 ± 0.03 mm, *n* = 22, *P* < 0.001; LVPW;d: 0.75 ± 0.02 mm, *n* = 22, *P* < 0.0001) and Nicotine + Ang-II mice (LVPW;s: 1.18 ± 0.03 mm, *n* = 19, *P* < 0.01; LVPW;d: 0.83 ± 0.03 mm, *n* = 19, *P* < 0.05). There were no significant differences in LVPW;s and LVPW;d between Nicotine + Ang-II mice and Nicotine mice (LVPW;s: 1.15 ± 0.02, *n* = 23; LVPW;d: 0.78 ± 0.02, *n* = 23) (Figures 1A,B). There were also no significant differences in LVID;s and LVID;s between any of the groups (Figures 1C,D).

These findings of impaired compensatory remodeling in a model of nicotine inhalation are consistent with studies using alternative nicotine exposure routes. Maternal nicotine exposure (100 μg/mL in drinking water during gestation) in rats reduces ejection fraction (EF), LVAW;s and LVPW;s in offspring *via* DNA methylation of cardiac-specific genes (40). Nicotine delivered by osmotic mini-pump at 6 mg/kg/day or 12 mg/kg/day in Sprague-Dawley and Long-Evans rats reduced heart length and heart weight, but did not change thickness of the LV, right ventricle (RV), or septal walls (41). Induction of pressure overload by transverse aortic constriction in ghrelin knockout mice induced elevations in heart weight indexed to tibial length, LVAW thickness, and LVPW thickness; treatment of this model with nicotine tartrate salt dissolved in the drinking water abrogated these changes (42). Rats subjected to LVAW myocardial infarction after 7-days of treatment with 1.75 mg/day of nicotine *via* patch were reported to have reduced LVAW thickness and increased dilatation of the LV chamber vs. mice that did not receive nicotine (43).

There was a main effect of Ang-II (*P* < 0.0001) to increase LVMI in both air-exposed and nicotine-exposed mice (Figure 1E). There was also a main effect of nicotine (*P* < 0.05) leading to a reduction of LVMI (Figure 1E). In contrast, RWT was increased in Air + Ang-II mice (*P* < 0.0001 vs. Air), but not Nicotine + Ang-II mice (*P* = 0.416 vs. Nicotine) (Figure 1F). These changes suggest three distinct groups: absent remodeling in Air and Nicotine mice, concentric hypertrophy in Air + Ang-II mice, and eccentric hypertrophy in Nicotine + Ang-II mice (44). Concentric and eccentric hypertrophy are both clinically correlated with HF (45). HF incidence is greater in patients with eccentric hypertrophy than in patients with concentric hypertrophy (45). Additionally, concentric hypertrophy is associated with relative preservation of cardiac function (HF with preserved EF, HFpEF) vs. eccentric hypertrophy (HF with reduced EF, HFrEF) (45). Matrix metalloproteinase (MMP) activation, leading to wall thinning and LV dilatation, is thought to play a mechanistic role in eccentric hypertrophy and HFrEF (44, 46, 47). Another study using osmotic delivery of both nicotine and Ang-II in mice reported increased MMP-2 activity, increased heart weight indexed to body weight, and dilatation of the aorta providing further support for our findings indicating a shift toward eccentric hypertrophy in mice exposed to inhaled nicotine while infused with Ang-II (48). Furthermore, cigarette smoke exposure upregulated MMP-9 and inhibited adaptive remodeling in a rat model of volume overload, leading to eccentric hypertrophy and impaired ventricular function, indicating overlap in the mechanisms of nicotine- and cigarette-induced pathology (49).

These findings, when considered in the context of LaPlace's Law, suggest that nicotine exposure induces a shift from concentric to eccentric hypertrophy during hemodynamic stress which may exacerbate LV wall stress and promote cardiac decompensation (Figure 1G). It is, however, important to acknowledge that Ang-II infusion is a model of hypertension and cardiac remodeling which does not progress to HF in mice. We found no significant differences in echocardiographic measurements of cardiac function (EF and fractional shortening, FS) between Air, Air + Ang-II, Nicotine, and Nicotine+Ang-II mice (Figures 1H,I). Future studies must explore additional models of hemodynamic stress which progress to cardiac dysfunction (such as transverse aortic constriction or myocardial ischemia-reperfusion injury) to better comprehend the role of nicotine in compensatory remodeling.

### Discussion

Our study and others provide evidence of impaired cardiac compensation in animal models of nicotine exposure. Nicotine is of particular interest as the primary biologically active component of novel tobacco products; however, emissions from these devices contain hundreds of additional chemicals creating complex interactions. At present, there have been no independent studies of the cardiac effects of heat-not-burn tobacco in animals. Studies conducted by researchers affiliated with Philip Morris International, a tobacco company and producer of the IQOS heat-not-burn system, reported reduced atherosclerosis, LV remodeling and dysfunction, and cardiac gene changes in female Apolipoprotein E knockout mice exposed to heat-not-burn aerosol vs. cigarette smoke (50, 51). Preclinical evidence of cardiac remodeling and dysfunction following electronic cigarette exposure is, in contrast, more robust (Table 1).

**Table 1:** Summary of animal studies demonstrating cardiac structural and functional changes associated with nicotine and novel tobacco products.

	**Ref**.	**Species**	**Model**	**Finding**
LV	N/A	C57BL/6 (Adult, ♂)	8 wks inhaled nic ± 4 wks Ang-II infusion	Concentric → Eccentric hypertrophy
	(40)	Sprague-Dawley (PND1, ♂/♀)	Nic in H_2_O of pregnant dams during gestation	↓ EF, ↓ LVAW, and LVPW in offspring
	(41)	Sprague-Dawley and Long-Evans (Adult, ♂/♀)	2 wks nic infusion	↓ HW, ↓ HL
	(42)	Ghrelin-KO C57BL/6 (Adult, ♂)	12 wks transverse aortic constriction ± nic tartrate salt in H_2_O	↓ HW, ↓ LVAW, and LVPW after transverse aortic constriction
	(43)	Sprague-Dawley (Adult, ♂)	1 wk nic patch + LVAW myocardial infarction	↓ LVAW, ↑ LV dilatation after myocardial infarction
	(48)	C57BL/6 (Adult, ♂)	4 wks nic infusion ± 4 wks ang II infusion	↑ HW, ↑ Cardiac MMP-2, ↑ Aortic dilatation
	(52)	ApoE-KO C57BL/6 (Adult, ♂)	12 wks e-cig (2.4% nic) inhalation	Cardiomyocyte abnormalities, ↓ EF and FS, ↑ ROS
	(53)	C57BL/6 (Adult, ♂)	12 wks e-cig (2.4% nic) inhalation ± HFD	Cardiomyocyte apoptosis, ↓ EF and FS, ↑ ROS
	(54)	C57BL/6 (Adult, ♂)	60 wks e-cig (24 mg/mL nic) inhalation	↑ HW, ↑ LVAW and LVPW, ↑ ROS, ↓ Vascular function
	(55, 56)	Sprague-Dawley (Adult, ♂)	4 wks nic intraperitoneal injection ± I/R and oral irbesartan	↑ Cardiac hypertrophy and fibrosis, ↑ infarct area, ↑ ROS, ↑ Inflammation, ↓ LV function after I/R attenuated by irbesartan
	(57)	FVB Mice (Adolescent and Adult, ♂/♀)	12 wks e-cig (20.2 mg/mL nic) inhalation	↑ Collagen I and III, ↓ LV systolic and diastolic function in adolescent ♂ only
RV	(32, 33)	C57BL/6 (Adult, ♂)	8 wks inhaled nic ± 8 wks losartan infusion	↑ RVSP, ↑ RV wall thickness, ↑ RV dilatation abrogated by losartan
	(58)	α7-nAChR-KO C57BL/6 (Adult, ♂/♀)	8-12 wks inhaled nic	↑ RVSP, ↑ RV wall thickness, ↑ RV dilatation, ↓ Vascular function in wildtype ♂ only
	(59)	C57BL/6 (Adult, ♂)	24 wks heated e-cig (24 mg/mL nic) inhalation	↑ RV wall thickness, ↓ RV function, ↑ Systemic inflammation

Ang, angiotensin; ApoE, Apolipoprotein E; E-cig, electronic cigarette; EF, ejection fraction; FS, fractional shortening; FVB, susceptible to Friend leukemia virus B; HFD, high-fat diet; HW and HL, heart weight and length; I/R, myocardial ischemia/reperfusion injury; LV, left ventricle; LVAW or PW, LV anterior or posterior wall thickness; MMP, matrix metalloproteinase; Nic, nicotine; nAChR, nicotinic acetylcholine receptor; PND, post-natal day; ROS, reactive oxygen species; RV, right ventricle; RVSP, RV systolic pressure.

Exposing Apolipoprotein E knockout mice to 12 weeks of electronic cigarette vapor containing 2.4% nicotine caused extensive cellular changes in cardiomyocytes including shrunken nuclei (indicative of apoptosis), abnormal myofibrils in the cytoplasm, mitophagy, mitochondrial DNA lesions, and accumulation of lipids and reactive oxygen species (52). These cardiomyocyte changes resulted in cardiac dysfunction including reduced FS and EF (52). C567BL/6J mice fed high fat diet and exposed to 12 weeks of electronic cigarette vapor with 2.4% nicotine showed reduced FS and EF associated with cardiomyopathy-like cellular changes on electron microscopy of the ventricle (53). Cardiomyocytes from mice exposed to electronic cigarette vapor with 2.4% nicotine exhibited increased apoptosis, increased oxidative stress, and reduced AMPK phosphorylation; these *in vivo* and *ex vivo* findings were absent in mice exposed to electronic cigarette vapor with 0% nicotine (53).

In C57BL/6J mice, 60 weeks of inhalation exposure to electronic cigarettes containing 24 mg/mL of nicotine resulted in thickening of the LVAW and LVPW which were comparable to 60 weeks of exposure to 3R4F research cigarettes (54). These changes in LV chamber dimensions were associated with increased heart weight indexed to body weight, superoxide production in the cardiac tissue, and cardiovascular dysfunction (54). Treatment of Sprague-Dawley rats with irbesartan, an Ang-II type 1 receptor (AT_1_R) antagonist, ameliorated cardiac hypertrophy, fibrosis, reactive oxygen species accumulation, inflammation, and LV dysfunction induced by 28 days of 0.6 mg/kg of nicotine delivered by intraperitoneal injection (55). This nicotine exposure model also exacerbated LV dysfunction and infarct size in Langendorff-perfused hearts undergoing *ex vivo* ischemia-reperfusion injury (55, 56). Three months of exposure to electronic cigarette vapor containing 20.2 mg/mL of nicotine in male, adolescent FVB (Susceptible to the Friend leukemia virus B) mice induced LV systolic and diastolic dysfunction which were not present in adult mice or female, adolescent mice (57).

In addition to changes in the LV, nicotine and novel tobacco products induce changes in pulmonary circulation and RV remodeling. We have previously shown that chronic, inhaled nicotine exposure causes pulmonary hypertension associated with RV remodeling, elevated RV brain-type natriuretic peptide, and increased expression of angiotensin-converting enzyme in the RV, but not LV (32). These changes are abrogated by treatment with losartan, a specific AT_1_R antagonist (33). Nicotine-induced pulmonary hypertension, RV remodeling, and vascular dysfunction are ameliorated by α7 nicotinic cholinergic receptor knockout, and are absent in female mice (58). The pathogenesis of pulmonary hypertension induced by Sugen and hypoxia also involves α7 nicotinic cholinergic receptor-mediated cross-talk between cardiomyocytes and cardiac fibroblasts, leading to RV fibrosis (60). Treatment of cardiac fibroblasts isolated from the RV, but not the LV, with cigarette smoke extracts or 600 nM of nicotine stimulated fibroblast proliferation (61). Mice exposed to 6 months of heated electronic cigarette liquid containing 24 mg/mL of nicotine developed significant RV wall thickening and RV dysfunction associated with extensive changes in systemic inflammation (59).

This study is limited by the inclusion of a single nicotine dose and exposure duration. The dose- and time-response relationships between nicotine and maladaptive cardiac remodeling have not been established. The effect of nicotine on other cardiovascular parameters (including heart rate, skin temperature, and blood pressure), however, exhibits a flattened response with moderate doses achieving maximal effects (62). An expanding number of studies, including ours, demonstrate nicotine-associated cardiac remodeling and dysfunction using numerous dosages, exposure durations, routes of administration, and animal models. This suggests complex relationships which warrant examination in future studies.

Cigarette smoking is strongly associated with cardiac remodeling and dysfunction (4–11). Early clinical studies of novel tobacco products have shown promising reductions in cardiovascular diseases when compared to cigarette smoking (23, 26, 27). Despite this, caution must be exercised due to the prolonged disease course of cardiovascular pathology in relation to the short history of novel tobacco products. Clinical studies using acute endpoints have reported cardiovascular changes which may predispose patients to the development of cardiac remodeling and dysfunction (14). Additionally, a growing body of preclinical evidence has linked nicotine and novel tobacco product exposure with significant cardiac remodeling and dysfunction in animal models. These findings are particularly concerning due to novel tobacco product use among young, never smokers which may lead to future cigarette smoking (63). Further exploration is necessary; however, clinicians and researchers should not overlook nicotine and novel tobacco product use as potential risk factors in the pathogenesis of cardiac remodeling and associated diseases including HF.

## Data availability statement

The raw data supporting the conclusions of this article will be made available by the authors, without undue reservation.

## Ethics statement

The animal study was reviewed and approved by LSUHSC IACUC.

## Author contributions

NF drafted the manuscript. EL, XY, and JG conceptualized experiments. NF and JO performed experiments and data analysis. NF and AW prepared figures. All authors edited the manuscript and approved final version.

## Funding

This study was supported by National Heart, Lung, and Blood Institute grants R01HL135635 and R01HL135635-S1 to EL, XY, and JG.

## Conflict of interest

The authors declare that the research was conducted in the absence of any commercial or financial relationships that could be construed as a potential conflict of interest.

## Publisher's note

All claims expressed in this article are solely those of the authors and do not necessarily represent those of their affiliated organizations, or those of the publisher, the editors and the reviewers. Any product that may be evaluated in this article, or claim that may be made by its manufacturer, is not guaranteed or endorsed by the publisher.
